# Turning to Religion During COVID-19 (Part II): A Systematic Review, Meta-analysis and Meta-regression of Studies on the Relationship between Religious Coping and Mental Health throughout COVID-19

**DOI:** 10.1007/s10943-022-01720-4

**Published:** 2023-01-03

**Authors:** Daniel Pankowski, Kinga Wytrychiewicz-Pankowska

**Affiliations:** 1 Faculty of Psychology, University of Warsaw, Stawki 5/7, 00-183 Warsaw, Poland; 2 University of Economics and Human Sciences in Warsaw, Warsaw, Poland

**Keywords:** Religious coping, COVID-19, Stress, Depression, Anxiety

## Abstract

The COVID-19 pandemic and the many associated socio-economic changes constitute a stressful event that required adaptation to new, dynamic, and often threatening conditions. According to the literature, coping strategies are one of the factors that determine a person’s degree of adaptation to stressful situations. A systematic review and meta-analysis was performed on the relationship between religious coping and selected indicators of mental health. Due to the large amount of data, this work has been divided into two parts: Part I discussed the positive mental health indicators (Pankowski & Wytrychiewicz-Pankowska, 2023), while this Part II discusses negative mental health indicators. A systematic review of the databases of Science Direct, EBSCO, Cochrane, PubMed, and Google Scholar identified 33 articles related to the severity of depressive symptoms: 30 to anxiety, 23 to stress, 1 related to PTSD symptoms and peritraumatic stress, and 5 related to general negative mental health. The limitations of the research as well as further directions for exploration are discussed.

*Clinical trial registration *This Review was pre-registered at OSF: osf.io/54ygr (https://doi.org/10.17605/OSF.IO/GMNFV).

## Introduction

Mental health (MH) issues are one of the greatest difficulties facing the healthcare system today (McCartan et al., 2021; Pfefferbaum & North, 2020). More and more studies and analyses are indicating the negative impact of mental health issues on, inter alia, work (Kessler et al., 2006), relationships (Sharabi et al., 2016), and other areas of life (Lépine & Briley, 2011). The COVID-19 pandemic has also led to the emergence and intensification of a variety of types of mental difficulties, through socio-economic factors (Agberotimi et al., 2020; Kourti et al., 2021; Lindau et al., 2021) as well as through psychological mechanisms (Cénat & Dalexis, 2020; Coelho et al., 2020).

Research conducted in various periods of the pandemic around the world has clearly shown the threat that it poses to mental well-being. Data from studies and reviews indicated a very high prevalence of, inter alia, depressive symptoms (Necho et al., 2021), anxiety (Kan et al., 2021), the presence of PTSD symptoms (Zhang et al., 2021), and peritraumatic stress (Jiménez et al., 2021). The above difficulties were the subject of research both in specific populations who were particularly exposed to stress resulting from, for example, the nature of their work (healthcare workers, in particular; Salari et al., 2020a), the risk of a more severe course of COVID-19 (Yan et al., 2022), but also the general population (Salari et al., 2020b). In addition to determining the scale of the problem, numerous analyses have focused on trying to identify factors that could buffer such negative responses to pandemic stress.

According to the theoretical models used in research on stress (Lazarus & Folkman, 1984; a more detailed description is provided in the first part of the review), coping strategies may be very important for MH in stressful situations. Earlier studies conducted in various populations confirmed the importance of coping strategies for MH, including the severity of depressive symptoms, anxiety, and stress. It should also be noted that coping strategies are a modifiable factor that can be shaped by interventions.

This part of the review focuses on negative mental health indicators: the relationships of religious coping (RC) with the severity of depressive symptoms, anxiety, stress levels, the severity of peri-/post-traumatic stress symptoms, and general MH indicators was analysed in more detail. The aim was to synthesize information from both cross-sectional and longitudinal studies and to try to determine the strengths of the relationships and the factors that may be responsible for variability in this area.

## Methods

Detailed data on search strategy, selection criteria, data extraction, quality assessment, and statistical analysis can be found in the first part of the review: Turning to Religion During COVID-19: A Systematic Review, Meta-analysis and Meta-regression of Studies on the Relationship between Religious Coping and Mental Health throughout COVID-19 (Part I) (Pankowski & Wytrychiewicz-Pankowska, 2023). Information on the quality of studies included in the review can be found in Appendix 1, funnel plots in Appendix 2 and number of studies and participants conducted per country in Appendix 3.

## Results

Detailed data on the review, including general information (number of people who participated in the research, broken down by country, etc.), can be found in the first part of the review; this part focuses on the description of studies describing the relationship between RC and negative MH indicators. Effect sizes obtained in meta-analyses were also transformed into Cohen’s *d*, *CLES* (Common Language Effect Size) and Odds ratio (Appendix 4).

### Severity of Depressive Symptoms and Religious Coping

First, the relationship between the severity of depressive symptoms and RC was analysed. A total of 33 articles analysing this relationship were identified. The research was conducted from November 2019 to August 2021 and a total of 24,644 people participated. Various methods were used to assess RC, including the Brief Coping Orientation to Problems Experienced Inventory (Brief-COPE), Brief Religious Coping Orientation to Problems Experienced Inventory (Brief-RCOPE), Spiritual/Religious Coping Scale (SRCOPE-14), and others. In the case of severity of depressive symptoms, various methods were also used: the Patient Health Questionnaire-9 (PHQ-9), Depression, Anxiety, and Stress Scale (DASS-21), and the Beck Depression Inventory (BDI). Almost half of the studies conducted indicated no relationship between RC and the severity of depressive symptoms. A summary of the results is shown in Table 1.

**Table 1:** Studies describing relationship between religious coping and severity of depressive symptoms

Authors [country]	Sample *N* [group]	Date started*	Date finished*	Basic sociodemographic characteristics	Tools used	Main findings
Albani et al. (2022) [Greece]	200 [nursing students]	1 March 2021	30 March 2021	86.5% of the sample were female; mean age was 22.8 years old (SD = 12.2)	RC: Brief-RCOPE; Depressive symptoms: HADS	pRC was not associated with depressive symptoms; nRC was positively associated with depressive symptoms
Alsolais et al. (2021) [Saudi Arabia]	492 [nursing students]	22 April 2020	16 May 2020	55.7% of the respondents were female; mean age was 21.77 years old (SD = 2.47)	RC: Brief-COPE; Depressive symptoms: DASS-21	RC was not a statistically significant predictor of depressive symptoms
Bakır et al. 2021 [Turkey]	327 [pregnant women]	1 July 2020	30 October 2020	100% of the sample were female; age: 17–25: 19.0%; 26–34: 49.5%; ≥ 35: 31.5%	RC: the scale developed by Abu-Raiya; Depressive symptoms: DASS-21	pRC was positively related to depression, while nRC was negatively related
Besirli et al. (2021) [Turkey]	200 [healthcare workers]	15 May 2020	15 June 2020	58.5% (*n* = 117) of the participants were female; mean age was 29.5 (SD = 6.4)	RC: COPE; Depressive symptoms: BDI	Negative correlation between RC and depressive symptoms
Budimir et al. (2021) [Austria]	1005 [GPs]	10 April 2020	30 April 2020	52.7% of the sample were female; n.i. about mean age	RC: SCI; Depressive symptoms: PHQ 9	No relationship between RC and depressive symptoms; linear regression analyses showed positive relationship of RC and depressive symptoms
Captari et al. (2022) [Colombia and South Africa]	1172 [Study 1: Colombian students] and 451 [Study 2: South Africans]	3 April 2020	25 May 2020	Women were 62.12% of the sample in Study 1 and 65.85% in Study 2. Mean age was 21.70 (SD = 3.96) in Study 1 and 33.54 (SD = 11.93) in Study 2	RC: RCOPE; Depressive symptoms: BSI18	pRC was associated negatively with depressive symptoms; the relationship between nRC and depression attenuated at higher levels of pRC among both gender groups
Chow et al. 2021 [Malaysia]	200 [healthcare workers]	n.i	n.i	60.5% of the sample were female; age: 20–30: 25.5%; 31–40: 70.5%; 41–50: 3.5%; > 51: 0.5%	RC: Brief-RCOPE; Depressive symptoms: HADS	nRC was positively correlated with depressive symptoms, while pRC was negatively correlated with depressive symptoms
Chui et al. 2021 [Malaysia]	859 [nurses]	1 April 2020	30 August 2020	n.i. about how many % of the sample were female; mean age was 32.7 (SD = 6.9)	RC: Brief-COPE; Depressive symptoms: MDI	No relationship between RC and depressive symptoms
Davis et al. (2021) [USA]	T1 (1 month prepandemic): 1036; T2 (1 month into the pandemic): 453; T3 (3 months into the pandemic): 302 [GPs]	6 February 2020	6 June 2020	Women were 47.4% of the sample; n.i. about age	RC: Brief-RCOPE; Depressive symptoms: PHQ-9	No relationship between RC and depressive symptoms
Faronbi et al. (2021) [Nigeria]	272 [nursing students]	1 November 2019	30 November 2019	Women were 89.0% of the group; mean age was 33.77 (SD = 5.71)	RC: Brief-COPE; Depressive symptoms: BDI	No relationship between RC and depressive symptoms
Fukase et al. (2022) [Japan]	1468 [GPs]	17 July 2020	22 July 2020	45.2% of the sample were female; mean age was 52.60 (SD = 15.82)	RC: Brief-COPE; Depressive symptoms: PHQ-9	No relationship between RC and depressive symptoms
Ghoncheh et al. (2021) [Iran]	696 [older adults]	1 November 2020	30 January 2021	57.9% of the sample were female; mean age was 69.56 years (SD = 9.31)	RC: SCS; Depressive symptoms: HADS	RC was negatively correlated with severity of depressive symptoms
Lopes and Nihei (2021) [Brasil]	1224 [undergraduate students]	14 September 2020	19 October 2020	68.6% of the sample were female; age: 18–24: 77.9%; > 24: 22.1%	RC: Brief-COPE; Depressive symptoms: DASS-21	RC was negatively correlated with depressive symptoms
Mahamid and Bdier (2021) [Palestine]	400 [GPs]	1 February 2020	26 February 2020	57% of the sample were female; age: 20–29: 45.3%; 30–39: 29.6%; 40–49: 15.5%; 50–59: 9.8%	RC: IPRC subscale of the Psychological Measure of Islamic Religiousness; Depressive symptoms: CES-D 10	pRC was negatively correlated with depressive symptoms
Margetić et al. (2022) [Croatia]	2860 [GPs]	4 April 2020	27 April 2020	80.6% of the sample were female; 18–24: 11.7%; 25–34: 27.4%; 35–44: 29.0%; 45–54: 20.6%; 55–64: 9.5%; 65 + : 1.8%	RC: WHOQoLSRPB; Depressive symptoms: DASS-21	RC was negatively associated with depressive symptoms
Mestas et al. (2021) [Mexico]	747 [GPs]	13 May 2020	28 May 2020	54.4% of the sample were female; mean age was 25.03 (SD = 8.95)	RC: SCQ Depressive symptoms: BDI	RC was negatively related to severity of depressive symptoms
Mishra et al. (2021) [India]	588 [medical, dental, and nursing students]	1 September 2020	30 October 2020	71.9% of the sample were female; mean age was 20.9 years (SD = 1.55)	RC: Brief-COPE; Depressive symptoms: DASS-21	RC was associated with a lower probability of having depression in the female subgroup
Narendra Kumar et al. (2022) [Malaysia]	173 [healthcare workers]	1 May 2021	31 August 2021	72.2% of the sample were female; mean age was 36.46 (SD = 8.05)	RC: Brief-COPE; Depressive symptoms: HADS	No relationship between RC and depressive symptoms
Park et al. (2021) [USA]	1015 [GPs]	7 April 2020	9 April 2020	Female was 53.9% of the sample; mean age was 38.9 years (SD = 13.50)	RC: Brief-COPE; Depressive symptoms: DASS-21	Depressive symptoms were negatively related with RC
Penengo et al. (2021) [Italy]	316 [pregnant women]	15 December 2020	15 June 2021	100% of the sample were female; mean age was 33.25 (SD = 5.24)	RC: Revised Prenatal Coping Inventory; Depressive symptoms: PHQ-2	No relationship between RC and depressive symptoms
Romdhane and Cheour (2021) [Tunisia]	603 [GPs]	9 April 2020	15 April 2020	Women consisted of 74.0% of the sample	Religious coping: A-BRCS; Depressive symptoms: DASS-21	nRC was positively correlated with depressive symptoms. pRC was negatively correlated with depressive symptoms. Regression analyses showed that nRC positively predicted depressive symptoms
Romero-García et al. (2022) [Spain]	434 [ICU Staff]	1 March 2020	30 June 2020	81.8% of the sample were female; The mean age was 41.33 years (SD = 9.80)	Religious coping: Brief-COPE; Depressive symptoms: PHQ-9	No relationship between RC and depressive symptoms
Rosa-Alcázar et al. (2021) [Spain]	122 [OCD patients] and 115 [healthy controls]	1 April 2020	30 April 2020	81.8% of the sample were female; mean age was 34.60 years (SD = 10.41)	Religious coping: Brief-COPE; Depressive symptoms: HADS	No relationship between RC and depressive symptoms
Shamblaw et al. (2021) [Canada]	T1: 797; T2 395 [GPs]	21 April 2020	27 May 2020	T1: 54.6% of the sample were female; The mean age was 32.2 years (SD = 11.5); T2: 55.7% of the sample were female; mean age was 33.7 years (SD = 12.6)	Religious coping: Brief-COPE; Depressive symptoms: PHQ9	No relationship between RC and depressive symptoms
Shehata et al. (2021) [Egypt]	283 [GPs]	20 May 2020	7 July 2020	74.2% of the sample were female; mean age was 34.81 years (SD = 11.36)	Religious coping: tool prepared by authors; Depressive symptoms: BDI-II	RC was negatively associated with depressive symptoms
Sitarz et al. (2021) [Poland]	2172 [students]	20 April 2020	26 April 2020	73% of the sample were female; mean age was 22.1 years (SD = 2.2)	Religious coping: Brief-COPE; Depressive symptoms: DASS-21	RC was negatively correlated with depressive symptoms
Smida et al. (2021) [Qatar]	127 [medical residents]	17 May 2020	16 June 2020	37% of the sample were female; n.i. about mean age	Religious coping: Brief-COPE; Depressive symptoms: DASS-21	No relationship between RC and depressive symptoms
Thomas and Barbato, (2020) [United Arab Emirates]	543 [GPs]	6 April 2020	17 April 2020	15,4% of the sample were female; mean age was 32.9 (SD = 11.10)	Religious coping: Brief-RCOPE; Depressive symptoms: PHQ-9	No relationship between RC and depressive symptoms
Umucu and Lee (2020) [USA]	269 [self-reported disabilities and chronic conditions]	1 April 2020	30 April 2020	43.9% of the sample were female; mean age was 39.37 years (SD = 12.18)	Religious coping: Brief-COPE; Depressive symptoms: PHQ-4	RC was no associated with depressive symptoms
Vitorino et al. (2021) [Brazil]	1156 [GPs]	11 May 2020	3 June 2020	69.6% of the sample were female; mean age was 37.6 years (SD = 14.0)	RC: SRCOPE-14; Depressive symptoms: PHQ-9	nRC was positively associated with depressive symptoms
Yee et al. (2021) [Malaysia]	528 [GPs]	1 April 2020	30 May 2020	n.i	RC: Brief-COPE; Depressive symptoms: DASS-21	RC was significantly associated with less mild-to-severe depression
Yıldırım et al. (2021) [Turkey]	259 [GPs]	n.i	n.i	88% of the sample were female; mean age was 32.96 years (SD = 8.88)	RC: Brief-RCOPE; Depressive symptoms: DASS-21	nRC was positively related to depression, while pRC was negatively associated with depressive symptoms
Zarrouq et al. (2021) [Morocco]	1435 [GPs]	3 April 2020	30 April 2020	43.0% of the sample were female; The mean age was 32.2 years (SD = 10.5)	RC: Brief-RCOPE; Depressive symptoms: HADS	nRC was positively associated with depressive symptoms

*RC* religious coping, *Brief-RCOPE* Brief Religious COPE, *HADS* The Hospital Anxiety and Depression Scale, *pRC* positive religious coping, *nRC* negative religious coping, *Brief-COPE* Brief Coping Orientation to Problems Experienced, *DASS-21* The Depression, Anxiety and Stress Scale, *BDI* Beck Depression Inventory, *GP* general population, *SCI* Stress and Coping Inventory, *PHQ* Patient Health Questionnaire, *BSI-18* Brief Symptom Inventory, *n.i.* no information, *MDI* major depression inventory, *T1* measurement 1, *T2* measurement 2, *T3* measurement 3, *SCS* The Spiritual Coping Strategy, *IPRC* Islamic Positive Religious Coping Scale, *CES-D* Center for Epidemiologic Studies Depression Scale, *WHOQoLSRPB* The WHO Quality of Life-Spirituality, Religiousness and Personal Beliefs, *SCQ* The Stress Coping Questionnaire, *A-BRCS* Arabic Brief religious Coping Scale, *SRCOPE-14* the Brief Scale for Spiritual/Religious Coping

*In studies that did not specify the exact dates on which data were collected (months only), the beginning (1) and end (30) of the month were used as the starting and ending points

For a more detailed analysis of the relationship between these two variables, we performed a meta-analysis. For inclusion of a measure in the analysis, we required that it appears in at least 3 surveys. A thorough analysis of the tools used allowed for the performance of a meta-analysis of the relationship between depressive symptoms assessed with DASS-21 and RC assessed with Brief-COPE (4 studies).

#### Meta-analysis

**Fig. 1:**
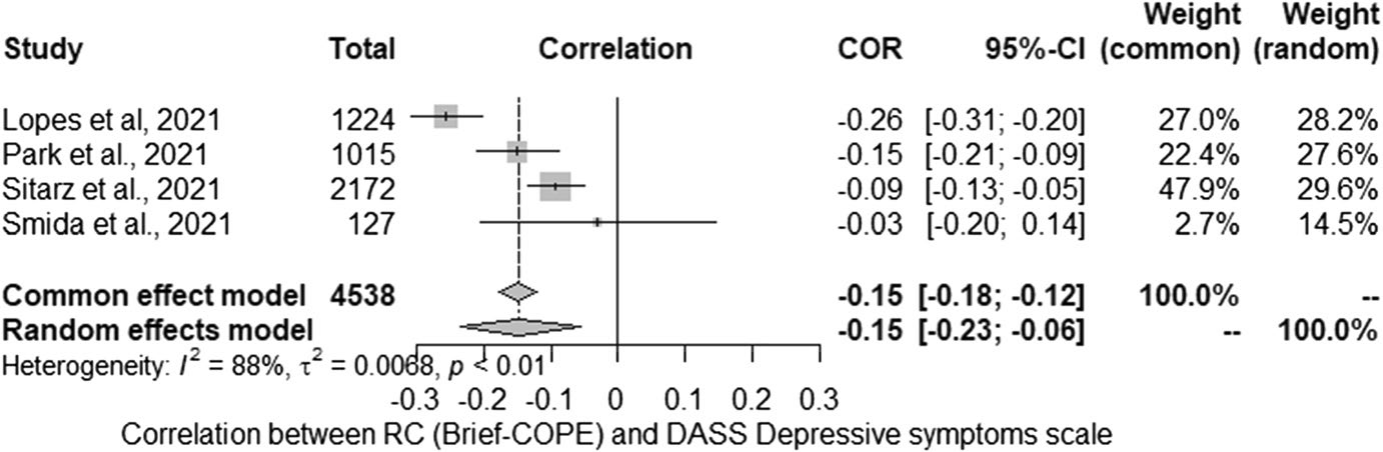
Religious coping and severity of depressive symptoms: forest plot

The meta-analysis conducted for the relationship between severity of depressive symptoms (DASS-21) and RC (Brief-COPE) included three studies. Studies identified in the literature search as meeting the inclusion criteria were pooled to give a correlation − 0.15 [− 0.23; − 0.06] which was statistically significant (*Z* =  − 3.18; *p* < 0.01) (Fig. 1). Statistically significant heterogeneity was observed between studies (*Q* = 24.01; *p* < 0.001). The estimated amount of total heterogeneity was Tau2 = 0.007 and *I*^2^ = 87.5%.

Due to the high heterogeneity of the results, the percentage of women was analysed as potential moderator. Unfortunately, due to deficiencies in the reported data, it was not possible to include more moderators. The tests for moderators showed that the percentages of women in the study (*QM* (1) = 0.55 non-significant) were not statistically significant moderator in the performed studies.

**Fig. 2:**
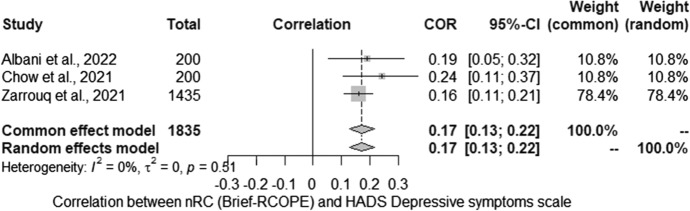
Relationship between negative religious coping and severity of depressive symptoms: forest plot

Next, the relationship between nRC and the severity of depressive symptoms (HADS) was analysed. The meta-analysis conducted for the relationship between severity of depressive symptoms (HADS) and nRC (Brief-RCOPE) included three studies. Studies identified in the literature search as meeting the inclusion criteria were pooled to give a correlation 0.17 [0.13; 0.22] which was statistically significant (*Z* = 7.44; *p* < 0.001) (Fig. 2). Statistically significant heterogeneity was not observed between studies (*Q* = 1.35; *p* > 0.05). The estimated amount of total heterogeneity was Tau2 = 0 and *I*^2^ = 0%.

**Fig. 3:**
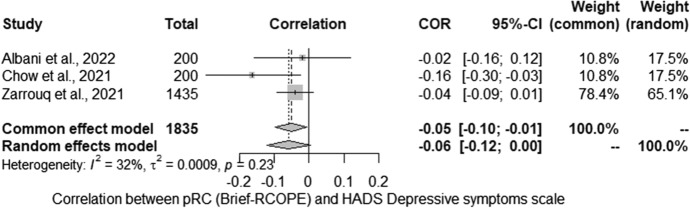
Relationship between positive religious coping and severity of depressive symptoms: forest plot

Then, the relationship between pRC and the severity of depressive symptoms (HADS) was analysed. The meta-analysis conducted for the relationship between severity of depressive symptoms (HADS) and pRC (Brief-RCOPE) included three studies. Studies identified in the literature search as meeting the inclusion criteria were pooled to give a correlation − 0.06 [− 0.12; 0.00] which was not statistically significant (*Z* =  − 1.81; *p* > 0.05) (Fig. 3). Statistically significant heterogeneity was not observed between studies (*Q* = 2.94; *p* > 0.05). The estimated amount of total heterogeneity was Tau2 = 0.0009 and *I*^2^ = 32.1%.

### Severity of Anxiety Symptoms and Religious Coping

Next, the relationship between the severity of anxiety symptoms and RC was analysed. A total of 30 studies analysing the relationship between anxiety and RC were identified. The research was conducted from February 2020 to August 2021 and a total of 21,368 people participated in it. Furthermore, a variety of methods were used to assess both anxiety and RC, including the Brief-COPE, Brief-RCOPE, SRCOPE-14, and others. Similarly for anxiety, a variety of methods were used, including DASS-21, HADS, and BAI. Almost half of the studies conducted indicated no relationship between RC and the severity of anxiety symptoms. A summary of the results is shown in Table 2.

**Table 2:** Studies describing the relationship between religious coping and severity of anxiety symptoms

Authors [country]	Sample *N* [group]	Date started*	Date finished*	Basic sociodemographic characteristics	Tools used	Main findings
Albani et al. (2022) [Greece]	200 [nursing students]	1 March 2021	30 March 2021	86.5% of the sample were female; mean age was 22.8 (SD = 12.2)	RC: Brief-RCOPE; Anxiety: HADS	pRC was not associated with anxiety; nRC was positively associated with anxiety
Alsolais et al. (2021) [Saudi Arabia]	492 [nursing students]	22 April 2020	16 May 2020	55.7% of the respondents were female; the mean age was 21.77 (SD = 2.47)	RC: Brief-COPE; Anxiety: DASS-21	RC was not a statistically significant predictor of anxiety
Bakır et al. (2021) [Turkey]	327 [pregnant women]	1 July 2020	30 October 2020	100% of the sample were female; age: 17–25: 19.0%; 26–34: 49.5%; ≥ 35: 31.5%	RC: the scale developed by Abu-Raiya; Anxiety: DASS-21	Anxiety was not associated with RC
Besirli et al. (2021) [Turkey]	200 [healthcare workers]	15 May 2020	15 June 2020	58.5% (*n* = 117) of the participants were female; mean age was 29.5 (SD = 6.4)	RC: COPE; Anxiety: BAI	No relationship between RC and anxiety
Budimir et al. (2021) [Austria]	1005 [GPs]	10 April, 2020	30 April, 2020	52.7% of the sample were female; n.i. about mean age	RC: SCI; Anxiety: GAD-7	Linear regression analyses showed positive relationship of RC with anxiety
Cansız et al. (2021) Turkey	1050 [frontline healthcare workers (*n* = 353); non-frontline healthcare workers (*n* = 347), 350 control group]	20 March 2020	10 April 2020	Women were: 68.28% of the control group, mean age = 35.71 (SD = 10.72); 61.38% of non-frontline healthcare workers, mean age = 34.26 (SD = 8.98); and 63.73% of frontline healthcare workers, mean age = 34.27 (SD = 7.70)	RC: Brief-COPE; Anxiety: STAI;	No relationship between anxiety and RC
Chow et al. (2021) [Malaysia]	200 [healthcare workers]	n.i	n.i	60.5% of the sample were female; age: 20–30: 25.5%; 31–40: 70.5%; 41–50: 3.5%; > 51: 0.5%	RC: Brief-RCOPE; Anxiety: HADS	nRC was positively correlated with anxiety while pRC was negatively correlated
Davis et al. (2021) [USA]	T1 (1 month prepandemic): 1036; T2 (1 month into the pandemic): 453; T3 (3 months into the pandemic): 302 [GPs]	6 February 2020	6 June 2020	Women were 47.4% of the sample; n.i. about age	RC: Brief-RCOPE; Anxiety: GAD-7	No relationship between RC and anxiety
Ghoncheh et al. (2021) [Iran]	696 [older adults]	1 November 2020	30 January 2021	57.9% of the sample were female; mean age was 69.56 years (SD = 9.31)	RC: SCS; Anxiety: HADS	RC was negatively correlated with severity of anxiety
Lopes and Nihei (2021) [Brasil]	1224 [undergraduate students]	14 September 2020	19 October 2020	68.6% of the sample were female; age: 18–24: 77.9%; > 24: 22.1%	RC: Brief-COPE; Anxiety: DASS-21	RC was negatively correlated with anxiety
Margetić et al. (2022) [Croatia]	2860 [GPs]	4 April 2020	27 April 2020	80.6% of the sample were female; 18–24: 11.7%; 25–34: 27.4%; 35–44: 29.0%; 45–54: 20.6%; 55–64: 9.5%; 65 + : 1.8%	RC: WHOQoLSRPB; Anxiety: DASS-21	RC was negatively associated with anxiety
Masha'al et al. (2022) [Jordan]	282 [nursing students]	n.i	n.i	74.1% of the sample were female; mean age was 20.08 (SD = 1.08)	RC: Brief-COPE; Anxiety: GAD-7	RC was not associated with anxiety
Mestas et al. (2021) [Mexico]	747 [GPs]	13 May 2020	28 May 2020	54.4% of the sample were female; mean age was 25.03 (SD = 8.95)	RC: SCQ; Anxiety: BAI	RC was negatively related with anxiety
Mishra et al. (2021) [India]	588 [medical, dental, and nursing students]	1 September 2020	30 October 2020	71.9% of the sample were female; mean age was 20.9 years (SD = 1.55)	RC: Brief-COPE; Anxiety: DASS-21	RC was not associated with anxiety
Narendra Kumar et al. (2022) [Malaysia]	173 [healthcare workers]	1 May 2021	31 August 2021	72.2% of the sample were female; mean age was 36.46 (SD = 8.05)	RC: Brief-COPE; Anxiety: HADS	RC reduced the level of anxiety
Park et al. (2021) [USA]	1015 [GPs]	7 April 2020	9 April 2020	Women were 53.9% of the sample; mean age was 38.9 years (SD = 13.50)	RC: Brief-COPE; Anxiety: DASS-21	No relationship between RC and anxiety
Penengo et al. (2021) [Italy]	316 [pregnant women]	15 December 2020	15 June 2021	100% of the sample were female; mean age was 33.25 (SD = 5.24)	RC: Revised Prenatal Coping Inventory; Anxiety: GAD-7	pRC was associated with greater anxiety
Quansah et al. (2022) [Ghana]	760 [physical education students]	n.i	n.i	26.5% of the sample were female; age: 20–24: 35.3%; 25–29: 16.3%; 30–34: 32.4% 35–39: 3.4%; > 40: 12.6%	RC: 16-item multidimensional scale by Quansah; Anxiety: adapted from the non-clinical symptoms of BAI	RC was not associated with anxiety
Romdhane and Cheour (2021) [Tunesia]	603 [GPs]	n.i	n.i	Women were 74.0% of the sample	RC: A-BRCS; Anxiety: DASS-21	nRC was positively correlated with anxiety. Regression analyses showed that nRC was a positive predictor of anxiety
Romero-García et al. (2022) [Spain]	434 [ICU staff]	1 March 2020	30 June 2020	81.8% of the sample were female; The mean age was 41.33 years (SD = 9.80)	RC: Brief-COPE; Anxiety: GAD-7	RC was not associated with anxiety
Rosa-Alcázar et al. (2021) [Spain]	122 [OCD patients] and 115 [healthy controls]	1 April 2020	30 April 2020	81.8% of the sample were female; The mean age was 34.60 years (SD = 10.41)	RC: Brief-COPE; Anxiety: HADS	RC was positively correlated with anxiety
Shamblaw et al. (2021) [Canada]	T1: 797; T2: 395 [GPs]	21 April 2020	27 May 2020	T1: 54.6% of the sample were female; the mean age was 32.2 years (SD = 11.5); T2: 55.7% of the sample were female; the mean age was 33.7 years (SD = 12.6)	RC: Brief-COPE; Anxiety: GAD-7	RC was not associated with anxiety
Shehata et al. (2021) [Egypt]	283 [GPs]	20 May 2020	7 July 2020	74.2% of the sample were female; the mean age was 34.81 years (SD = 11.36)	RC: tool prepared by authors; Anxiety: STAI	RC was negatively associated with anxiety
Sitarz et al. (2021) [Poland]	2172 [students]	20 April 2020	26 April 2020	73% of the sample were female; the mean age was 22.1 years (SD = 2.2)	RC: Brief-COPE; Anxiety: DASS-21	RC was positively correlated with anxiety
Smida et al. (2021) [Qatar]	127 [medical residents]	17 May 2020	16 June 2020	37% of the sample were female; n.i. about mean age	RC: Brief-COPE; Anxiety: DASS-21	No relationship between RC and anxiety
Thomas and Barbato (2020) [United Arab Emirates]	543 [GP]	6 April 2020	17 April 2020	15.4% of the sample were female; mean age was 32.9 (SD = 11.10)	RC: Brief-RCOPE; Anxiety: GAD-7	RC was not related with anxiety
Vitorino et al. (2021). [Brazil]	1156 [GP]	11 May 2020	3 June 2020	69.6% of the sample were female; The mean age was 37.6 years (SD = 14.0)	RC: SRCOPE-14; Anxiety: GAD-7	nRC was positively associated with anxiety pRC was positively associated with anxiety
Williams et al. (2021) [Australia]	151 [students]	1 April 2020	30 April 2020	74.8% female; n.i. about mean age	RC: Brief-COPE; Anxiety: GAD-7	RC was positively related with anxiety
Yıldırım et al. (2021) [Turkey]	259 [GP]	n.i	n.i	88% of the sample were female; the mean age was 32.96 years (SD = 8.88)	RC: Brief-RCOPE; Anxiety: DASS-21	nRC was positively related to anxiety
Zarrouq et al. (2021) [Morocco]	1435 [GP]	3 April 2020	30 April 2020	43.0% of the sample were female; the mean age was 32.2 years (SD = 10.5)	RC: Brief-RCOPE; Anxiety: HADS	nRC was positively associated with anxiety; pRC was positively associated with anxiety

*RC* religious coping, *Brief-RCOPE* Brief Religious COPE, *HADS* The Hospital Anxiety and Depression Scale, *pRC* positive religious coping, *nRC* negative religious coping, *Brief-COPE* Brief Coping Orientation to Problems Experienced, *DASS-21* The Depression, Anxiety and Stress Scale, *BAI* Beck Anxiety Inventory, *GP* general population, *SCI* Stress and Coping Inventory, *GAD-7* generalized anxiety disorder, *STAI* State-Trait Anxiety Inventory, *n.i.* no information, *SCS* The Spiritual Coping Strategy, *WHOQoLSRPB* The WHO Quality of Life-Spirituality, Religiousness and Personal Beliefs, *SCQ* The Stress Coping Questionnaire, *A-BRCS* Arabic Brief religious Coping Scale, *SRCOPE-14* the Brief Scale for Spiritual/Religious Coping

*In studies that did not specify the exact dates on which data were collected (months only), the beginning (1) and end (30) of the month were used as the starting and ending points

#### Meta-analysis

The analysis of the studies included in the review allowed us to perform three meta-analyses concerning the relationship between RC assessed with Brief-COPE and Brief-RCOPE and the severity of anxiety assessed with HADS.

**Fig. 4:**
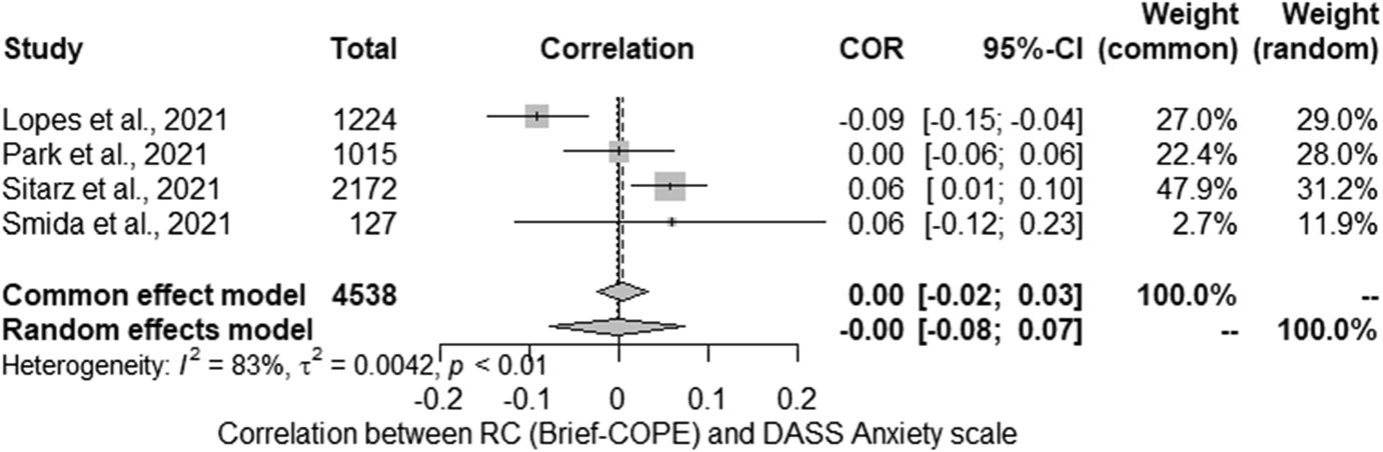
Religious coping assessed with Brief-COPE and anxiety: forest plot

The meta-analysis conducted for the relationship between severity of anxiety (DASS-21) and RC (Brief-COPE) included four studies. Studies identified in the literature search as meeting the inclusion criteria were pooled to give a correlation 0.00 [− 0.08; 0.07] which was not statistically significant (*Z* =  − 0.04; *p* > 0.05) (Fig. 4). Statistically significant heterogeneity was observed between studies (*Q* = 17.58; *p* < 0.01). The estimated amount of total heterogeneity was Tau2 = 0.04 and *I*^2^ = 83%.

Due to the high heterogeneity of the results, the percentage of women was analysed as potential moderator. Unfortunately, due to deficiencies in the reported data, it was not possible to include more moderators. The tests for moderators showed that the percentage of women in the study (*QM* (1) = 0.12; non-significant) was not statistically significant moderator.

**Fig. 5:**
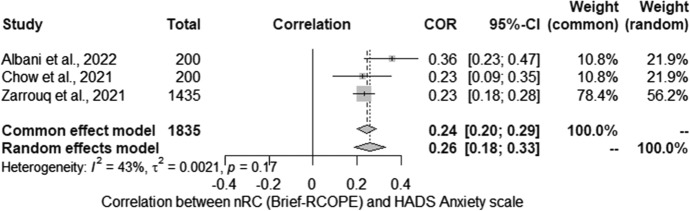
Negative religious coping and anxiety: forest plot

Subsequently, the relationship between nRC and the severity of anxiety assessed with HADS was analysed. The meta-analysis conducted for the relationship between severity of anxiety (HADS) and nRC (Brief-RCOPE) included three studies. Studies identified in the literature search as meeting the inclusion criteria were pooled to give a correlation 0.26 [0.18; 0.33] which was statistically significant (*Z* = 6.66; *p* < 0.001) (Fig. 5). Statistically significant heterogeneity was not observed between studies (*Q* = 3.50; *p* > 0.05). The estimated amount of total heterogeneity was Tau2 = 0.002 and *I*^2^ = 43%.

Potential moderators were also analysed: the percentage of women and relationship status (the percentage of married people). Unfortunately, due to deficiencies in the reported data, it was not possible to include more moderators. The tests for moderators showed that the percentage of women in the study (*QM* (1) = 2.88; *p* = 0.09) and the percentage of married people (*QM* (1) = 2.98; *p* = 0.08) were at the statistical trend level.

**Fig. 6:**
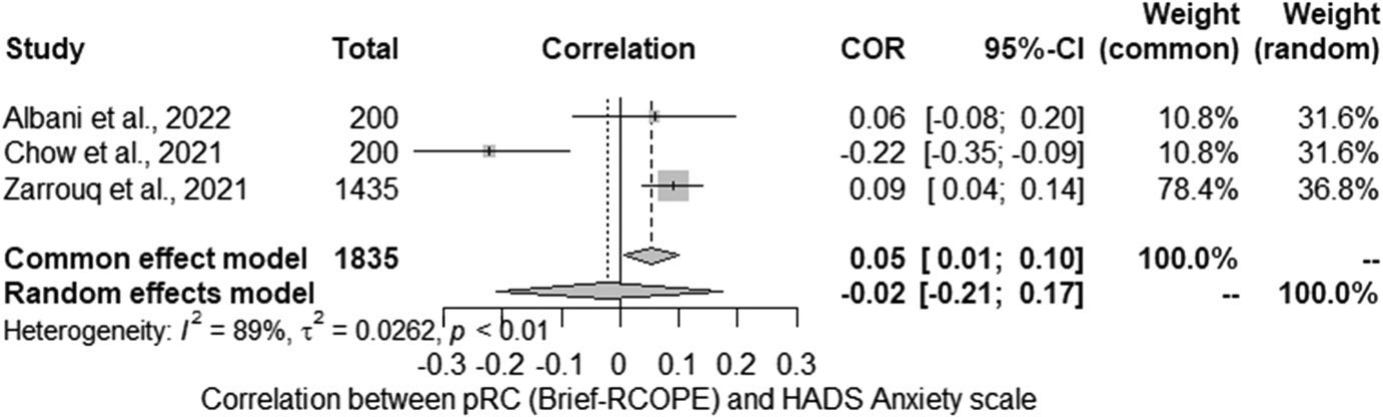
Positive religious coping and anxiety: forest plot

Finally, we performed a meta-analysis concerning the relationship between pRC and levels of anxiety. The meta-analysis conducted for the relationship between severity of anxiety (HADS) and pRC (Brief-RCOPE) included three studies. Studies identified in the literature search as meeting the inclusion criteria were pooled to give a correlation − 0.02 [− 0.21; 0.17] which was not statistically significant (*Z* =  − 0.21; *p* > 0.05) (Fig. 6). Statistically significant heterogeneity was observed between studies (*Q* = 17.53; *p* < 0.01). The estimated amount of total heterogeneity was Tau2 = 0.03 and *I*^2^ = 87%.

Due to the high heterogeneity of the results, potential moderators were also analysed in more detail: the percentage of women and relationship status (the percentage of married people). The tests for moderators showed that the percentage of women in the study (*QM* (1) = 0.00; non-significant) and the percentage of married persons (*QM* (1) = 0.81; non-significant) were not statistically significant.

### Stress

In the next step, the relationship between stress level and RC was analysed. A total of 23 studies meeting the criteria were identified. The research was conducted from March 2020 to October 2020. A total of 16,557 people participated in the research. In the vast majority of studies, the Brief-COPE was used to assess RC, and stress levels were measured with the Perceived Stress Scale (PSS) and DASS-21. In over half of the studies (12), no significant statistical relationship was found between RC and the level of stress. A summary of the results is shown in Table 3.

**Table 3:** Studies describing the relationship between religious coping and level of stress

Authors [country]	Sample *N* [group]	Date started*	Date finished*	Basic sociodemographic characteristics	Tools used	Main findings
Alsolais et al. (2021) [Saudi Arabia]	492 [nursing students]	22 April 2020	16 May 2020	55.7% of the respondents were female; the mean age was 21.77 years old (SD = 2.47)	RC: Brief-COPE; Stress: DASS-21	RC was not a statistically significant predictor of stress
Awoke et al. (2021) [Ethiopia]	337 [health science students]	1 August 2020	5 September 2020	48.4% participants were female; mean age of the participants was 22.88 (SD = 1.78) years	RC: Brief-COPE; Stress: PSS-10	RC was significantly associated with perceived stress level in both bivariate and multivariate regression analyses
Babore et al. (2020) [Italy]	595 [healthcare workers]	11 April 2020	16 April 2020	80.3% of the sample were women; mean age of 40.69 years (SD = 11.48)	RC: COPE NVI 25; Stress: PSS	No relationship between stress and RC
Bakır et al. (2021) [Turkey]	327 [pregnant women]	1 July 2020	30 October 2020	100% of the sample were female; age: 17–25: 19.0%; 26–34: 49.5%; ≥ 35: 31.5%	RC: the scale developed by Abu-Raiya; Stress: DASS-21	RC was not related to stress
Besirli et al. (2021) [Turkey]	200 [healthcare workers]	15 May 2020	15 June 2020	58.5% (*n* = 117) of the participants were female; mean age was 29.5 (SD = 6.4)	RC: COPE; Stress: PSS-10	No relationship between stress and RC
Bianchi et al. (2021) [Italy]	1929 [GPs]	1 April 2020	30 May 2020	71.6% of the sample were female; mean age was 24.17 (SD = 2.75)	RC: COPE NVI 60; Stress: Pandemic-Related Stress	Weak positive relationship between RC and pandemic-related stress
Budimir et al. (2021) [Austria]	1005 [GPs]	10 April 2020	30 April 2020	52.7% of the sample were female; n.i. about mean age	RC: SCI; Stress: PSS-10	Linear regression analyses showed positive relationship of RC and stress
Chui et al. (2021) [Malaysia]	859 [nurses]	n.i	n.i	n.i. about % of women in sample; mean age was 32.7 (SD = 6.9)	RC: Brief-COPE; Stress: PSS	No relationship between stress and RC
El Tahir et al. (2022) [Qatar]	100 [families of adults with intellectual disabilities]	7 June 2020	7 September 2020	52% of the sample were women; n.i. about mean age	RC: Brief-COPE; Stress: PSS	No relationship between stress and RC
Girma et al. (2021) [Ethiopia]	613 [adults with chronic diseases]	1 March 2020	30 March 2020	38.2% of the sample were female; mean age was 36.93 years (SD = 1.68)	RC: Brief-COPE; Stress: PSS	RC was positively correlated with stress level
Lopes and Nihei (2021) [Brasil]	1224 [undergraduate students]	14 September 2020	19 October 2020	68.6% of the sample were female; age: 18–24: 77.9%; > 24: 22.1%	RC: Brief-COPE; Stress: DASS-21	RC was negatively correlated with stress
Mahamid and Bdier (2021) [Palestine]	400 [GPs]	1 February 2020	26 February 2020	57% of the sample were female; age: 20–29: 45.3%; 30–39: 29.6%; 40–49: 15.5%; 50–59: 9.8%	RC: IPRC subscale of the Psychological Measure of Islamic Religiousness; Stress: PSS	pRC was negatively correlated with stress
Margetić et al. (2022) [Croatia]	2860 [GPs]	4 April 2020	27 April 2020	80.6% of the sample were female; 18–24: 11.7%; 25–34: 27.4%; 35–44: 29.0%; 45–54: 20.6%; 55–64: 9.5%; 65 + : 1.8%	RC: WHOQoLSRPB; Stress: DASS-21	RC was negatively associated with stress
Mishra et al. (2021) [India]	588 [medical, dental, and nursing students]	1 September 2020	30 October 2020	71.9% of the sample were female; mean age was 20.9 years (SD = 1.55)	RC: Brief-COPE; Stress: DASS-21	No relationship between stress and RC
Park et al. (2021) [USA]	1015 [GPs]	7 April 2020	9 April 2020	Women was 53.9% of the sample; mean age was 38.9 years (SD = 13.50)	RC: Brief-COPE; Stress: DASS-21	Stress level was negatively related with RC
Romdhane and Cheour (2021) [Tunisia]	603 [GPs]	9 April 2020	15 April 2020	Women were 74.0% of the sample; n.i. about mean age	RC: A-BRCS; Stress: DASS-21	nRC was positively correlated with stress. pRC was negatively correlated with stress
Sitarz et al. (2021) [Poland]	2172 [students]	20 April 2020	26 April 2020	73% of the sample were female; the mean age was 22.1 years (SD = 2.2)	RC: Brief-COPE; Stress: DASS-21	No relationship between stress and RC
Smida et al. (2021) [Qatar]	127 [medical residents]	17 May 2020	16 June 2020	37% of the sample were female; n.i. about mean age	RC: Brief-COPE; Stress: DASS-21	No relationship between RC and stress
Umucu and Lee (2020) [USA]	269 [self-reported disabilities and chronic conditions]	1 April 2020	30 April 2020	43.9% of the sample were female; the mean age was 39.37 years (SD = 12.18)	RC: Brief-COPE; Stress: PSQ-8	RC was positively associated with stress
Vannini et al. (2021) [USA]	141 [older adults]	07 May, 2020	26 May, 2020	58.87% of the sample were female; the mean age was 74.36 years (SD = 8.35)	RC: Brief-COPE; Stress: PSS-14	No relationship between stress and RC
Willey et al. (2022) [USA]	176 [older adults]	23 March 2021	13 May 2021	58% of the sample were female; mean age was 76.3 (SD = 8.94)	RC: Brief-COPE; Stress: PSS	No relationship between stress and RC
Yeung et al. (2022) [Hong Kong]	266 [Filipina domestic helpers]	9 May 2020	17 May 2020	n.i. about % of women in sample; age: 18–25: 0.8%;26–35: 36.1%;36–45: 41.3%; 46–55: 17.3%; > 55: 3.7%	RC: Brief-COPE; Stress two items, on a 5-point scale, ranging from “not at all true” to “always true”	No relationship between RC and COVID-19 stress
Yıldırım et al. (2021) [Turkey]	259 [GPs]	n.i	n.i	88% of the sample were female; the mean age was 32.96 years (SD = 8.88)	RC: Brief-RCOPE; Stress: DASS-21	nRC was positively related to stress, while pRC was negatively associated with stress

*RC* religious coping, *Brief-COPE* Brief Coping Orientation to Problems Experienced, *DASS-21* The Depression, Anxiety and Stress Scale, *PSS-10* perceived stress scale, *GP* general population, *SCI* Stress and Coping Inventory, *n.i.* no information, *IPRC* Islamic Positive Religious Coping Scale, *pRC* positive religious coping, *nRC* negative religious coping, *WHOQoLSRPB* The WHO Quality of Life-Spirituality, Religiousness and Personal Beliefs, *A-BRCS* Arabic Brief religious Coping Scale, *PSQ* Perceived Stress Questionnaire, *Brief-RCOPE* Brief Religious COPE

*In studies that did not specify the exact dates on which data were collected (months only), the beginning (1) and end (30) of the month were used as the starting and ending points

#### Meta-analysis

Analysis of the research allowed us to conduct one meta-analysis regarding the relationship between RC assessed with Brief-COPE and the intensity of stress assessed with DASS-21. Unfortunately, despite the large number of studies assessing the relationship between stress measured with PSS and RC, it was not possible to obtain the data needed to perform the calculations (correlation coefficients).

**Fig. 7:**
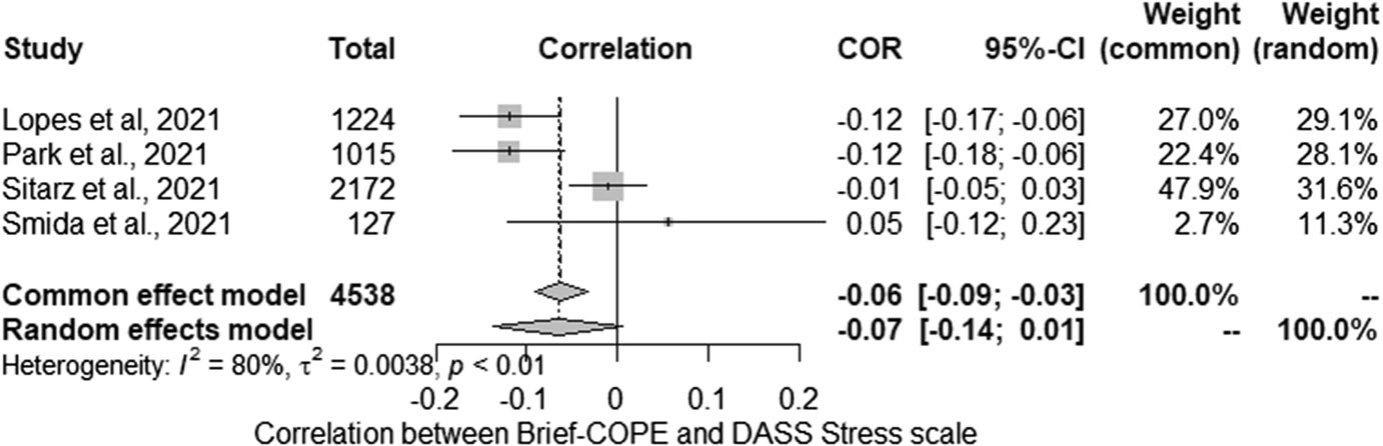
Religious coping and level of stress: forest plot

The meta-analysis conducted for the relationship between level of stress (DASS-21) and RC (Brief-COPE) included four studies. Studies identified in the literature search as meeting the inclusion criteria were pooled to give a correlation − 0.06 [− 0.09; − 0.03] which was not statistically significant (*Z* =  − 1.80; *p* > 0.05) (Fig. 7). Statistically significant heterogeneity was observed between studies (*Q* = 14.86; *p* < 0.01). The estimated amount of total heterogeneity was Tau2 = 0.04 and *I*^2^ = 80%.

Due to the high heterogeneity of the results, the percentage of women was analysed as potential moderator. The tests for moderators showed that the percentage of women in the study (*QM* (1) = 0.16; non-significant) was not statistically significant moderator of the conducted research.

### Peri- and Post-traumatic Stress Disorder Symptoms and General Mental Health Indicators

Lastly, the strength of the relationship between RC and the severity of peri- (*n* = 1) and post-traumatic (*n* = 1) stress disorder and general mental health indicators (*n* = 5) was analysed. The research was conducted from March 2020 to June 2020. In the RC assessment studies, Brief-COPE and Brief-RCOPE were mainly used. A variety of tools were used to assess negative mental health indicators: HADS, DASS, and GHQ-12 overall scores. A summary of the results is shown in Table 4. Due to the large variety of tools used, it was not possible to perform a meta-analysis.

**Table 4:** Religious coping and peri- and post-traumatic stress disorder symptoms and general mental health indicators

Authors [country]	Sample *N* [group]	Date started*	Date finished*	Basic sociodemographic characteristics	Tools used	Main findings
Peri- and post-traumatic stress disorder symptoms						
Park et al. (2021) [USA]	1015 [GPs]	7 April 2020	9 April 2020	Women was 53.9% of the sample; mean age was 38.9 years (SD = 13.50)	RC: Brief-COPE; Peritraumatic Distress Inventory	No relationship between RC and peritraumatic stress symptoms
Vancappel et al. (2021) [France]	1010 [healthcare workers]	24 March 2020	28 June 2020	83% of the sample were female; the mean age was 39.24 years (SD = 11.13)	RC: Brief-COPE; IES-6	RC was positively related with PTSD symptoms
General mental health indicators						
Anjum et al. 2022 [Pakistan]	320 [diagnosed with corona virus and they were in quarantine]	n.i	n.i	45.31% of the sample were women; mean age was 36.5 (SD = 5.6)	RC: Religious Coping Scale; Mental health: DASS-21	RC was positively related with DASS-21 global score
Eisenbeck et al. (2022) [Algeria, Argentina, Australia, Bangladesh, Brazil, Canada, Colombia, Egypt, France, Germany, Hungary, India, Indonesia, Italy, Lebanon, Mexico, New Zealand, Nigeria, Pakistan, Poland, Portugal, Romania, Russia, Slovenia, Spain, Sweden, Thailand, Turkey, UK, USA]	11,227 [GPs]	1 March 2020	30 June 2020	69.9% of the sample were women; mean age was 35.36 (SD = 13.26)	RC: Brief-COPE; Stress: Mental health: DASS-21	RC was positively correlated with psychological distress
Jarego et al. (2021) [Portugal]	430 [GPs]	1 April 2020	2 May 2020	71% of the sample were female; mean age was 39.9 (SD = 14.44)	RC: Brief-COPE; Mental health: MHI-5	RC was not related with mental health
Kandeğer et al. (2021) [Turkey]	84 [patients admitted to the COVID-19 inpatient clinic]	1 April 2020	1 June 2020	44% of the sample were female; mean age was 36.7 (SD = 3.1)	RC: Brief-RCOPE; Mental health: HADS	RC was not correlated with HADS
Rahimi Che et al. (2021) Malaysia	450 [students]	1 March 2020	30 June 2020	81.% of the sample were female; mean age was 21.85 (SD = 1.89)	RC: Brief-RCOPE; Mental health: GHQ-12	pRC was negatively correlated with mental health, while nRC was positively correlated

*RC* religious coping, *Brief-COPE* Brief Coping Orientation to Problems Experienced, *IES* Impact Event Scale, *PTSD* Post-traumatic Stress disorder, *n.i.* no information, *DASS-21* The Depression, Anxiety and Stress Scale, *GP* general population, *MHI* Mental Health Inventory, *Brief-RCOPE* Brief Religious COPE, *HADS* The Hospital Anxiety and Depression Scale, *GHQ* general health questionnaire, *pRC* positive religious coping, *nRC* negative religious coping

*In studies that did not specify the exact dates on which data were collected (months only), the beginning (1) and end (30) of the month were used as the starting and ending points

## Discussion

This second part of the systematic review focused on the relationship between Religious Coping (RC) and negative mental health (MH) indicators: severity of depressive symptoms, anxiety, stress, symptoms of peri- and post-traumatic stress disorder, and general negative indicators of MH. The vast majority of studies were cross-sectional and approximately half of them indicated no relationship between RC and the analysed variables. Despite the large number of studies included in the review, few could be analysed further. The meta-analyses performed included only 3–4 studies and therefore should be interpreted with caution. For meta-analysis of studies using Brief-COPE, in which RC is operationalized in a neutral manner, the results were characterized by statistically significant heterogeneity, which could not be explained with the use of possible moderators. Conversely, meta-analyses of the relationships of positive RC (pRC) and negative RC (nRC) with negative MH indexes.

The vast majority of studies on the relationship between RC and the severity of depressive symptoms were cross-sectional. Data were collected in different countries, at different stages of the pandemic, and in diverse populations. Our attempt to synthesize the results shows that they are very inconclusive: about half of the studies indicate no relationship, which is also confirmed by the results of two longitudinal studies; many analyses also indicate that this relationship is negative. We further explored this relationship with the use of meta-analyses, which indicated negative correlation (Brief-COPE), positive correlation (nRC) and no relationship (pRC) between the analysed variables. The first of them, concerning the neutral RC (Brief-COPE), was characterized by a very high heterogeneity of the results, while none of the possible moderators turned out to be statistically significant. As in the first part of the systematic review, attention should be paid to both the method of reporting the results (in the form of a correlation matrix) and the description of the studied sample, which were very different between the analysed studies. Further calculations with nRC and pRC showed a positive and lack of relationship, respectively, with MH. In the case of these analyses, the results were homogeneous, but they were based on very little data (*n* = 3), which significantly limits the possibility of generalizing these results. To sum up, it is worth considering a similar direction of dependence as in the case of QoL (see first part of the review), which suggested a greater influence of mood on the strategy chosen than the influence of a given strategy on the severity of depressive symptoms. At this point, it is worth emphasizing that the content of the items contained in the Brief-RCOPE (p/nRC) may reflect the effects of the coping process to a greater extent than the respondents’ approaches to stressful situations (see also first part of review).

Regarding levels of anxiety and RC, the results of the studies are very similar to those obtained for intensification of depressive symptoms, which may be due to the strong correlation between these two variables. The studies included in the review were conducted on a wide variety of populations, in many countries, and using a variety of methodologies. Longitudinal studies showed no correlation between RC and anxiety, as did a large proportion of cross-sectional studies. The meta-analysis of studies assessing the relationship between Brief-COPE and the level of anxiety found no relationship, but it was characterized by high heterogeneity of the results which could not be explained by moderators. In turn, nRC was characterized by a positive relationship with anxiety levels, and the result was homogeneous. This result should be interpreted with caution due to the small number of studies included. In the case of pRC, large heterogeneity of the results was noted: some results indicated a positive relationship, others a negative relationship. Again, the moderators that could be considered did not explain this variation between surveys. It should be noted that the meta-analyses of the relationship between RC and anxiety included the same studies as in the case of the intensification of depressive symptoms.

Next, the relationship between stress levels and RC was analysed. As before, the studies included in the review were conducted in many countries, populations, and throughout the pandemic period. The different methodology used to assess RC and stress made it difficult to more accurately analyse the relationship between the variables. All studies included in the review were cross-sectional, and half of them indicated no relationship between the analysed variables. Due to the ambiguous results of our investigation, it was decided to carry out a meta-analysis, which also indicated a very large diversity in the results. The statistically significant heterogeneity was not explained by the moderators used in the meta-regression. Summing up, the analysis of studies identified by the review indicated no relationship or a very weak relationship between RC and levels of stress, which perhaps suggests that RC may have a different function, not necessarily related only to stress reduction.

Lastly, studies on peri- and post-traumatic stress and general negative MH indicators were analysed. In the case of the first two variables, we found only one study each; it is therefore not possible to generalize these results. No relationship was found with peritraumatic stress disorder, and a study of post-traumatic stress disorder symptoms showed a positive relationship. On the other hand, studies on the relationship between RC and general negative MH indicators partially indicated a positive relationship in studies that used DASS-21, but no relationship for studies that used MHI-5 and HADS. In the case of the above studies, it was not possible to analyse the results in more depth, and the small number of studies makes it difficult to generalize. In conclusion, studies on the relationship between RC and negative mental health indicators suggest that this strategy has little or no protective effect. Moreover, some studies even indicate negative mental health effects associated with RC. In view of the positive relationship between RC and PTSD, it should also be considered to what extent the relationship between RC and PTG suggested in first part of review may be due to spiritual bypassing (see: Cashwell et al., 2010). It should be emphasized that only one study on the relationship between PTSD and RC could be identified; therefore, the relationships of RC with PTG, PTSD, and spiritual bypassing require further in-depth analysis. As indicated in the first part of the review, most analyses focused on the role of a given strategy in the variable-centred approach; it is possible that RC may be effective in a specific group of people or in a specific configuration of the strategies used, but it would require more in-depth analysis to confirm this.

### Study Limitations

Similarly, as noted in the first part of the review, the cross-sectional nature of most of the analysed studies prevents conclusions about the impact of RC on MH. The results show the frequency of using a specific strategy with the simultaneous level of MH indicators and not the effect of the coping process. The conducted review also has several limitations. The most important is the small number of studies included in the meta-analyses, especially when the same studies were analysed for different MH indicators. In addition, as noted earlier, inconsistent descriptions of sample structures and the absence of correlation coefficients between the analysed variables significantly limited possibilities for further data analysis. Thus, we appeal to authors to consider the variables describing the studied population as broadly as possible: not only age or gender, but also others that may be relevant, because the obtained results may differ radically in studies conducted on different populations. Furthermore, reporting correlation coefficients between the variables examined would facilitate further meta-analysis of results. Unfortunately, due to missing data, it was not possible to perform a network meta-analysis.

## Conclusions

The collected longitudinal data suggest that religious coping is not an effective method of coping with stress caused by the pandemic, especially in terms of negative mental health indicators. Data from cross-sectional studies suggest that this strategy, in particular negative coping, is used in connection with increased depressive or anxiety symptoms. A large proportion of studies failed to find any relationship between these two variables, as did individual longitudinal studies. It is possible that RC may play a different role that was not considered in the review, such as reducing the fear of death (Freh & Cheung Chung, 2021).

## Figures and Tables

**Fig. 8:**
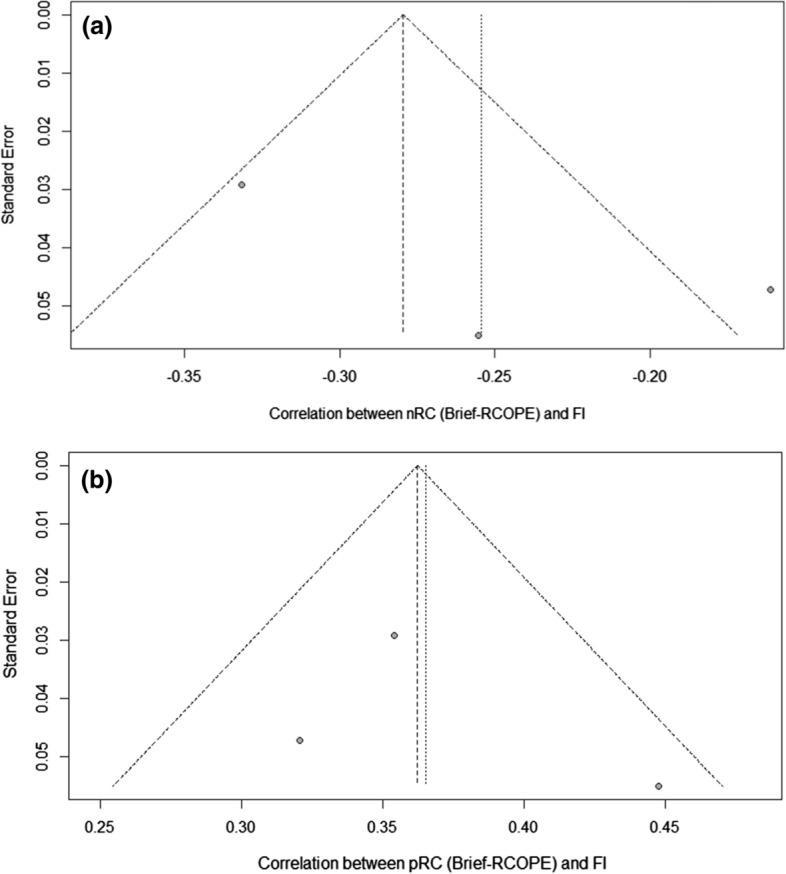
**a** Negative religious coping and flourishing: funnel plot. **b** Relationship between flourishing and positive religious coping: forest plot

**Fig. 9:**
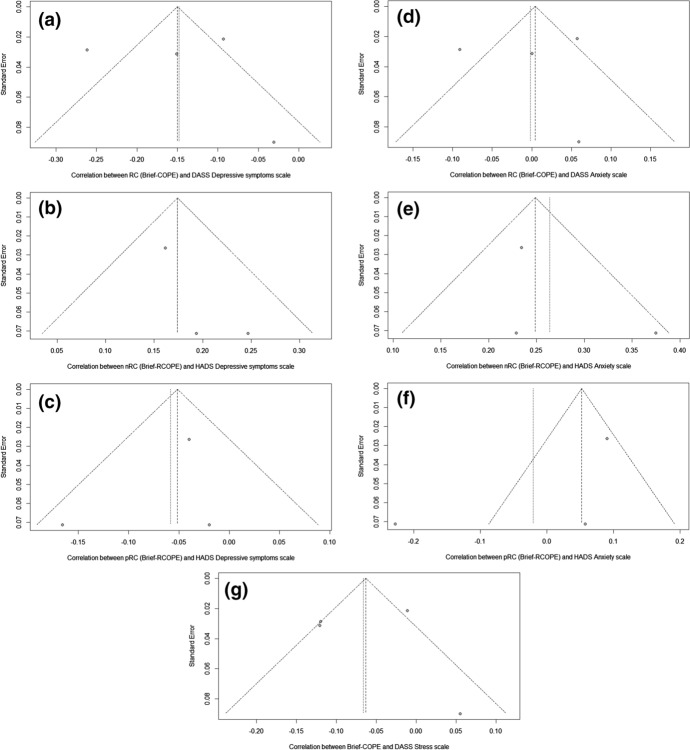
**a** Religious coping and severity of depressive symptoms: funnel plot. **b** Relationship between negative religious coping and severity of depressive symptoms: funnel plot. **c** Relationship between positive religious coping and severity of depressive symptoms: funnel plot. **d** Religious coping assessed with Brief-COPE and anxiety: funnel plot. **e** Negative religious coping and anxiety: forest plot. **f** Positive religious coping and anxiety: forest plot. **g** religious coping and level of stress: forest plot

## Data Availability

Data supporting findings are available at: osf.io/54ygr (https://doi.org/10.17605/OSF.IO/GMNFV).

## Appendix 1

**Table:**

Authors [country]	Selection				Comparability	Outcome		Sum
	1	2	3	4	1	1	2	
Albani et al. (2022)	1	0	0	2	1	1	1	6
Alsolais et al. (2021)	1	1	0	2	1	1	1	7
Altunan et al. (2021)	1	1	0	2	1	1	1	7
Anjum et al. (2022)	1	0	0	2	1	1	0	5
Awoke et al. (2021)	1	1	0	2	2	1	1	8
Babore et al. (2020)	1	1	0	2	2	1	1	8
Bakır et al. (2021)	1	1	0	2	1	1	1	7
Besirli et al. (2021)	1	0	0	2	1	1	1	6
Bianchi et al. (2021)	1	1	0	2	2	1	1	8
Budimir et al. (2021)	1	1	0	2	1	1	1	7
Cansız et al. (2021)	1	1	0	2	1	1	1	7
Captari et al. (2022)	1	0	0	2	2	1	1	7
Chow et al. (2021)	1	0	0	2	2	1	1	7
Chui et al. (2021)	1	1	0	2	2	1	1	8
Counted et al. (2022)	1	0	0	2	2	1	1	7
Davis et al. (2021)	1	1	0	2	2	1	1	8
Dobrakowski et al. (2021)	1	0	0	2	2	1	1	7
Eisenbeck et al. (2022)	1	1	0	2	2	1	1	8
El Tahir et al. (2022)	1	0	0	2	1	1	1	6
Faronbi et al. (2021)	1	1	0	2	1	1	1	7
Fukase et al. (2022)	1	1	0	2	1	1	1	7
Ghoncheh et al. (2021)	1	1	0	2	2	1	1	8
Girma et al. (2021)	1	1	0	2	1	1	1	7
Gupta et al. (2022)	1	0	0	2	1	1	1	6
Habib et al. (2020)	1	0	0	2	1	1	1	6
Jarego et al. (2021)	1	1	0	2	2	1	1	8
Kandeğer et al. (2021)	1	0	0	2	1	1	1	6
Lopes and Nihei (2021)	1	1	0	2	2	1	1	8
MacIntyre et al. (2020)	1	0	0	2	2	1	1	7
Mahamid and Bdier (2021	1	0	0	2	1	1	1	6
Margetić et al. (2022)	1	1	0	2	2	1	1	8
Masha'al et al. (2022)	1	0	0	2	1	1	1	6
Menculini et al. (2021)	1	1	0	2	1	1	1	7
Mestas et al. (2021)	1	0	0	2	2	1	1	7
Mishra et al. (2021)	1	1	0	2	2	1	1	8
Moussa et al. (2022)	1	1	0	2	1	1	1	7
Narendra et al. (2022)	1	1	0	2	2	1	1	8
Park et al. (2021)	1	1	0	2	2	1	1	8
Penengo et al. (2021)	1	1	0	2	2	1	1	8
Quansah et al. (2022)	1	0	0	2	2	1	1	7
Rahimi Che et al. (2021)	1	1	0	2	2	1	1	8
Romdhane and Cheour (2021)	1	0	0	2	2	1	1	7
Romero-García et al. (2022)	1	1	0	2	1	1	1	7
Rosa-Alcázar et al. (2021)	1	0	0	2	2	1	1	7
Shamblaw et al. (2021)	1	1	0	2	2	1	1	8
Shehata et al. (2021)	1	0	0	2	1	1	1	6
Sitarz et al. (2021)	1	0	0	2	1	1	1	6
Smida et al. (2021)	1	0	0	2	1	1	1	6
Thomas et al. (2020)	1	0	0	2	1	1	1	6
Umucu et al. (2020)	1	0	0	2	1	1	1	6
Vancappel et al. (2021)	1	0	0	2	2	1	1	7
Vannini et al. (2021)	1	0	0	2	2	1	1	7
Vitorino et al. (2021)	1	0	0	2	2	1	1	7
Willey et al. (2022)	1	0	1	2	2	1	1	8
Williams et al. (2021)	1	0	0	2	2	1	1	7
Yee et al. (2021)	1	1	0	2	2	1	1	8
Yeung et al. (2022)	1	1	0	2	2	1	1	8
Yıldırım et al. (2021)	1	0	0	2	2	1	1	7
Zarrouq et al. (2021)	1	1	0	2	1	1	1	7

## Appendix 2: Funnel Plots of Analysed Studies

The results should be interpreted with caution due to the small number of studies (Figs. 8a, b, 9a–g).

## Appendix 3

See Table

**Table 5:** Number of studies and participants conducted per country

	Number of studies	Participants
Algeria	1	253
Argentina	1	145
Australia	2	204
Austria	1	1005
Bangladesh	1	344
Brazil	3	2678
Canada	2	1129
Colombia	3	2456
Croatia	1	2860
Egypt	2	568
Ethiopia	2	950
France	2	1475
Germany	1	281
Ghana	1	760
Greece	1	200
Hong Kong	1	266
Hungary	1	262
India	2	1184
Indonesia	1	277
Iran	1	696
Italy	5	24,071
Japan	1	1468
Jordan	1	282
Lebanon	2	627
Malaysia	5	2210
Mexico	2	1395
Morocco	1	1435
New Zealand	1	43
Nigeria	2	707
Pakistan	3	940
Palestine	1	400
Poland	3	2812
Portugal	2	913
Qatar	2	227
Romania	1	546
Russia	1	307
Saudi Arabia	2	592
Slovenia	1	1271
South Africa	2	902
Spain	3	1196
Sweden	1	278
Thailand	1	405
Tunesia	1	603
Turkey	7	2427
UK	1	382
United Arab Emirates	1	543
USA	6	2918

^5^.

## Appendix 4

See Table

**Table 6:** Transformation of the effect sizes

Variables	*r*	Cohen's *d*	CLES	Odds ratio
Turning to religion during COVID-19: a systematic review, meta-analysis and meta-regression of studies on the relationship between religious coping and mental health throughout COVID-19 (Part I)				
Flourishing and nRC (Brief-RCOPE)	− 0.25	− 0.52	41.96%	0.39
Flourishing and pRC (Brief-RCOPE)	0.35	0.75	61.38%	3.88
Turning to religion during COVID-19: a systematic review, meta-analysis and meta-regression of studies on the relationship between religious coping and mental health throughout COVID-19 (Part II)				
Severity of depressive symptoms (DASS-21) and RC (Brief-COPE)	− 0.15	− 0.30	45.21%	0.58
Severity of depressive symptoms (HADS) and nRC (Brief-RCOPE)	0.17	0.35	55.44%	1.87
Severity of depressive symptoms (HADS) and pRC (Brief-RCOPE)	− 0.06	− 0.12	48.09%	0.80
Severity of anxiety (DASS-21) and RC (Brief-COPE)	0	0	50%	1
Severity of anxiety symptoms (HADS) and nRC (Brief-RCOPE)	0.26	0.54	58.37%	2.66
Severity of anxiety symptoms (HADS) and pRC (Brief-RCOPE)	− 0.02	− 0.04	49.36%	0.93
Level of stress (DASS-21) and RC (Brief-COPE)	− 0.06	− 0.12	48.09%	0.80

*CLES* Common Language Effect Size, *nRC* negative religious coping, *Brief-RCOPE* Brief Religious COPE, *pRC* positive religious coping, *DASS-21* The Depression, Anxiety and Stress Scale, *Brief-COPE* Brief Coping Orientation to Problems Experienced, *HADS* The Hospital Anxiety and Depression Scale

6.
